# Disability‐based arguments against assisted dying laws

**DOI:** 10.1111/bioe.13036

**Published:** 2022-04-07

**Authors:** Ben Colburn

**Affiliations:** ^1^ Department of Philosophy, University of Glasgow, Glasgow United Kingdom of Great Britain and Northern Ireland

**Keywords:** assisted dying, autonomy, criminal law, disability, euthanasia

## Abstract

Some of the most common arguments against legalizing assisted dying are based on appealing to the rights of people with disabilities. This article identifies and responds to those arguments, including that people with disabilities univocally oppose assisted dying laws; that those laws harm people with disabilities, or show disrespect; and that those laws undermine other vital aspects of healthcare. Drawing on philosophical argument, as well as on evidence from jurisdictions where assisted dying is legal, the article concludes that considerations of disability do not in fact generate good arguments against assisted dying laws. In fact, the opposite is true. There are nevertheless important lessons that proponents and defenders of such laws can learn in conversation with people with disabilities, including about safeguards on assisted dying to protect their well‐being and autonomy.

## 1 INTRODUCTION

Some of the most common arguments against the proposal to legalize assisted dying are based on appealing to the rights of people with disabilities.^1^ This article identifies and responds to several such arguments, concluding that considerations of disability do not in fact generate good arguments against legalizing assisted dying. There are nevertheless important lessons that proponents of change can learn from reflection on the perspectives of people with disabilities, including about safeguards on assisted dying to protect their well‐being and autonomy.

## 2 DO PEOPLE WITH DISABILITIES OPPOSE ASSISTED DYING LAWS?

Opponents of assisted dying laws often assert or imply that there is a consensus amongst people with disabilities that assisted dying should be prohibited,^2^ and the ‘monolithic’ opposition of disability rights organizations is often assumed even in academic research.^3^ In reality, this picture of unanimity is not borne out by the evidence.

A recent survey of disability rights organizations in the U.K. indicated varied stances on this matter.^4^Of 140 such organizations surveyed, a substantial majority either remained silent (84%) or explicitly endorsed neutrality (4%) on assisted dying. Only 4% explicitly opposed it. For those who remained neutral, the position of Disability Rights U.K. is representative: ‘This is a complex issue on which people hold different, passionately held views. Disability Rights UK respects those different views’.^5^ In many cases, the same reasoning is likely to hold for those who remained silent.

If we move from considering disability rights organizations to the positions of people with disabilities themselves, then the picture is even more mixed. As Tom Shakespeare observes, ‘notwithstanding the blanket opposition of “their” organisations, people with disabilities in the United Kingdom do not oppose assisted dying with one voice… at a minimum the views of the wider community are more mixed than the views of their leaders’.^6^ In fact, polling suggests strong support for assisted dying laws amongst people with disabilities, albeit with concerns.^7^ Other studies suggest that the level of support from people with disabilities for assisted dying laws is roughly the same as that in the general population.^8^

The evidence is therefore that people with disabilities, and disability rights organizations, have diverse views on the question of whether assisted dying should be legal. So, it is wrong for campaigners against assisted dying to assert that disabled people are univocally fearful or opposed. At best, such assertions are emphatic expressions of the convictions only of individual people with disabilities;^9^ at worst, they look like morally dubious attempts at misrepresentation.^10^ Either way, the overgeneralization shows disrespect, and does not take the full spectrum of the perspectives of people with disabilities seriously.

What does it mean to show genuine respect, and to take disabled perspectives seriously? The lived experience of people with disabilities is a vital source of wisdom and warning about what it means to live with vulnerabilities, and how those vulnerabilities can interact with wider social and economic pressures to put people under duress when making important decisions about their own lives. The best way forward for assisted dying campaigners is to engage with people with disabilities to see where they themselves see risks and problems, and then to make a robust judgment about what those factors require: do they undermine the case for assisted dying, or are they best understood as feeding into the design of safeguards?

The survey mentioned above of U.K. disability rights organizations identified the following reasons offered for opposing assisted dying laws.

1. Palliative care services are currently inadequate.
2. Genuinely autonomous choices are not currently possible.
3. Issue requires further research.
4. Disabled people may be directly pressured into opting for assisted dying.
5. Disabled people may be indirectly pressured into assisted dying.
6. Any safeguarding measures will be ineffective or open to abuse.
7. Medical decisions are unreliable and often inconsistent.
8. A ‘slippery slope’ will result in widening of coverage.
9. The policy focus should be on care and support rather than on assisted dying.
10. Assisted dying values profit more than people.
11. It is never right to help someone to die.
12. Assisted dying is unnecessary.
13. Assisted dying reflects the prejudices that disabled people face.
14. Assisted dying would further devalue the lives of disabled people.
15. Disabled people are encouraged to give up.
16. Assisted dying would undermine trust in healthcare.
17. The doctor–patient relationship would be harmed.
18. Legalization is widely opposed.
19. Legalization fails to respect the sanctity of life, and suicide should not be aided.
20. Legalization introduces inequity among disabled people.^11^

We can set some of these aside. (3) is answered by this article and the extensive research it reports. (11) and (19) are dogmatic expressions of individual religious/moral beliefs. (12) begs the question, since whether or not assisted dying is necessary is precisely the debate. (18) is disproved, as shown above. (20) is not an argument against legalization, but a complaint that a particular proposed law would be discriminatory by not extending the right to assisted dying to all people with disabilities.^12^ The rest can be summarised with the following questions, slightly reordered to aid clarity:

A. Do assisted dying laws harm people with disabilities? (6, 7, 8)
B. Do assisted dying laws undermine people with disabilities' autonomy? (1, 2, 4, 5, 9)
C. Are people with disabilities disrespected by assisted dying laws? (13, 14, 15)
D. Will legalization damage healthcare for people with disabilities? (1, 9, 10, 16, 17)

The remainder of this article answers these questions in turn. Section 2 considers Question A, and summarizes the body of international evidence about the impact of assisted dying laws on people with disabilities. Section 3 considers Questions B and C together, since—as we will see—the questions of disrespect for disabled lives and of concern for disabled peoples' autonomy are very closely linked. Section 4 answers Question D by looking at evidence about the impact of assisted dying on other parts of the healthcare system that are important for people with disabilities.

## 3 DO ASSISTED DYING LAWS HARM PEOPLE WITH DISABILITIES?

Opposition to assisted dying laws often focuses on the idea that such laws are especially harmful to people with disabilities, that safeguards inevitably fail, and that there will be a ‘slippery slope’ from apparently rigorous protections to loose and harmful practices.^13^

It is interesting, given how often the argument appears, that it tends not to be based on evidence, but made from the philosophical armchair, without other support save, for example, that the author finds that this causal claim is ‘at least plausible’^14^ or seems ‘more likely than not’.^15^ On its own terms, such speculation proves nothing. It does express real and understandable fears about assisted dying laws, and those fears generate hypotheses for testing against real evidence, with a view to both evaluating proposed safeguards for assisted dying laws, and securing legitimacy and confidence within the disabled community. However, these fears are not themselves evidence that assisted dying laws disproportionately impact people with disabilities. And when the hypotheses they inspire *are* tested, they are not borne out by the facts.

Let us start by summarizing three systematic reviews published between 2012 and 2016, which captured all the then‐published data (since legalization in each jurisdiction) on the uptake of assisted dying amongst vulnerable people, including people with disabilities.

Rietjens et al. explored the characteristics of cases, including by looking for a correlation between vulnerability (including disability) and uptake of assisted dying.^16^ They found evidence of different *kinds* of end‐of‐life decisions between different demographic groups, but the only strong correlation they found in uptake was with higher levels of education. They considered the hypothesis that ‘due to legalisation the rates of euthanasia would increase in “vulnerable” patient groups’ and concluded that ‘there is no clear evidence for a slippery slope’ of this kind.^17^

Steck et al. analysed all published studies to draw out the socio‐demographic characteristics of cases of assisted dying.^18^ They found some patterns (e.g., in most jurisdictions the majority of cases were male, and there was a correlation with higher education and secular beliefs) but no correlation with vulnerability in general or with disability specifically.

Emanuel et al. summarized the data from all jurisdictions with assisted dying at that point: Belgium, Canada, Colombia, Luxembourg, the Netherlands, and five U.S. states.^19^ They concluded that ‘In no jurisdiction was there evidence that vulnerable patients have been receiving euthanasia or physician‐assisted suicide at rates higher than in the general population … data do not indicate widespread abuses of these practices’,^20^ and that the hypothesis that people with disabilities might be disproportionately impacted ‘does not seem to be borne out’.^21^

This conclusion is reinforced if we look directly at the empirical data, some of which was included in the three reviews just mentioned, and some of which has been published since.

One study, which focused on Oregon and the Netherlands, found that ‘there is no current evidence for the claim that [assisted dying] will have disproportionate impact on patients in vulnerable groups’.^22^ In fact, vulnerable patients were under‐represented. The study also explored various specific dimensions of vulnerability. They found no evidence of heightened risk for people with disabilities and non‐terminal conditions,^23^ and ‘no current factual support for so‐called slippery‐slope concerns about the risks of legalisation of assisted dying’.^24^ Some commentators have criticized this study, but their worries are about under‐estimating the impact on groups other than people with disabilities.^25^ There is anyway reason to think that those criticisms are confused, for example because they take evidence of a higher‐than‐average proportion of cases from some groups, and mistakenly infer that the given group is at higher risk. In fact, the evidence just shows that there is bigger uptake in some groups; this is better explained by greater need and individual choice, rather than by differences in risk.

Another study compared cases of assisted dying in Belgium in 2007 and 2013.^26^ This showed a significant increase in requests, but found no disproportionate impact on people with disabilities, nor^27^ an especial increase in requests made by or granted to people with disabilities.^28^ The study attributed the shift to ‘continuing attitudinal and cultural shifts; [and because] values of autonomy and self‐determination have become more prominent, and acceptance of euthanasia continues to increase in the population at large’.^29^

These findings—that there is no evidence that assisted dying laws have a disproportionate effect on people with disabilities—are echoed in all empirical studies that examine the question. In the Netherlands, a study looking for problems for people with disabilities (alongside other factors) identified nothing, and instead found ‘evidence that the day‐to‐day practice of euthanasia discussions is much more conducive to maintaining life than previously understood’.^30^ A similar Dutch study likewise found no correlation between disability and uptake of assisted dying.^31^ A landmark survey of 20 years of evidence from Oregon found the same thing there.^32^ In Belgium, a study found that ‘[t]he repeatedly expressed concern that vulnerable people (older people, disabled people, those with psychiatric disorders) would more easily receive euthanasia is not supported by our data’.^33^ In Switzerland, a study noted some cases of people with severe disabilities seeking assisted death (as is legal under Swiss law), but identified nothing problematic about this—these cases were not evidence of greater vulnerability for people with disabilities—save that reporting requirements could benefit from being clarified.^34^ In Canada there is comparatively little evidence yet, and nothing that specifically examines people with disabilities; but there is data that shows that—as elsewhere—uptake of assisted dying is not correlated with socio‐economic disadvantage.^35^

More research would be useful, in particular to exclude the risk of peripheral or indirect effects of legalization for people with disabilities. But the fact that there is no evidence to support claims of disproportionate impact is very revealing, given this point's centrality to many disability‐based arguments against assisted dying law. Its absence significantly undermines those arguments.

## 4 DO ASSISTED DYING LAWS DISRESPECT PEOPLE WITH DISABILITIES?

A second theme is that assisted dying laws are disrespectful to people with disabilities, in particular by communicating that disabled lives are not worth living, or at any rate are less worth living than other lives are.^36^ Perpetuating such stereotypes is bad in itself. It also relates to the third theme I identified above, about autonomy: it is often claimed that assisted dying laws lead to people with disabilities being ‘coerced, manipulated, or forced’ to seek assisted dying.^37^ The two topics are connected because the internalization of negative stereotypes is a key mechanism whereby the autonomy of people with disabilities is purportedly undermined, and also because whether or not people with disabilities are coerced by assisted dying laws hinges on the substantive question of what respect for disabled lives and autonomy requires.

The argument from disrespect is mistaken in two ways. The first is that it misreports the core of the argument *for* assisted dying, which is not that some lives are less worth living than others, but rather that each individual must decide what makes their life worth living, whether it remains so in their own eyes, and what to do about it, including seeking an assisted death if that is what they judge to be right for themselves.^38^ This principle—of equal respect for each individual's autonomy—involves no comparative judgments about how worthwhile different lives are. Indeed, it arguably rules out such judgments.^39^ In the words of Baroness Hale, one of the law lords who heard the appeal in the important U.K. case of *R (Purdy) v DPP*:

it is not for society to tell people what to value about their own lives … If we are serious about protecting autonomy we have to accept that autonomous individuals have different views about what makes their lives worth living.^40^

The second mistake is more serious: this line of argument against assisted dying is in fact itself guilty of exactly the sin it ascribes to that position, namely of showing disrespect. As Christopher Riddle puts it, ‘Denying people with disabilities the right to exercise autonomy over their own life and death says powerfully damaging things about the disabled, their abilities, and their need to be protected’.^41^ Riddle goes on to argue that we best show respect by legalizing assisted dying while also ‘denying the dominant view that people with disabilities are pitiable individuals, lacking the critical thinking skills required to assess the value of their own lives when weighed against suffering at the end of life’.^42^

This point—that opponents of assisted dying thereby show disrespect for the individual lives and autonomy of people with disabilities—crops up repeatedly. It is voiced by people with disabilities themselves. Participants in one study ‘often expressed concern that disabled people may be especially vulnerable to being denied end‐of‐life choices because of the way they are devalued in society’, and that ‘people with disabilities may be denied choice because they are assumed incompetent to make their own decision’.^43^ Interviewees in another expressed the view that this stance was ‘discriminatory against people with disabilities’.^44^ This concern is also articulated in the academic literature, with scholars arguing that opposing assisted dying laws on these grounds treats people with disabilities ‘as some anonymous “disabled person” lacking a character’^45^ and ‘as incompetent, easily coerced, and inclined to end their lives places them in the roles to which they have been confined by disability discrimination’.^46^ Many people with disabilities ‘find this stereotyping to be itself demeaning and patronising, complaining that it feeds rather than starves social prejudices’.^47^ Even some opponents of assisted dying acknowledge the danger of a ‘paternalistic over‐emphasis on the vulnerability of persons with disabilities’.^48^

There are undoubtedly challenges, especially for determining whether capacity and consent are present in cases where cognitive functioning or communicative capacity is impeded.^49^ But a blanket prohibition on assisted dying is the wrong way to respond to those challenges. That relies on a pejorative stereotype and ignores the ways that appropriate support can facilitate autonomous decision‐making for people with intellectual disabilities.^50^

All interlocutors in these debates clearly want to show respect for people with disabilities. There is a shared determination to counteract stereotypes, for example that people with disabilities lack capacity to make significant decisions on their own behalf, or that their lives lack value compared with those of their non‐disabled peers. What people disagree about is what these shared concerns demand in terms of law. It is a matter of controversy how best to show respect, and whether assisted dying confounds or confirms harmful stereotypes. In the face of such deep and conscientious disagreement, the appropriately neutral stance is to remove the legal prohibition, and let individual citizens decide the difficult ethical questions for themselves.^51^

## 5 DOES ASSISTED DYING DAMAGE HEALTH AND SOCIAL CARE FOR PEOPLE WITH DISABILITIES?

The fourth theme identified in Section 1 concerns the effects of assisted dying laws on the healthcare system. This section addresses the impact on palliative care provision and the consequences for the doctor–patient relationship.

The worry that assisted dying laws will undermine funding or support for palliative care is widely expressed.^52^ It features frequently in submissions from medical organizations when new legislation is proposed. For example, Dr. Francis Dunn of the Royal College of Physicians in Glasgow, reporting on behalf of that body to a committee of the Scottish Parliament in 2015, said that:

if assisted suicide was an option, it could affect other options, such as further development of the palliative care movement. If [assisted suicide] had come in 20 years ago, it would have diminished the impetus for the palliative care movement. There are still many further developments that could be made in palliative care, particularly for non‐malignant conditions. If assisted suicide were an option on the table, it would not be possible to explore the other options in the same way. That is a real issue.^53^

These worries are not borne out by evidence from jurisdictions with assisted dying laws. In fact, assisted dying tends to go hand in hand with greater support for palliative care, financially and otherwise. In Belgium, assisted dying ‘is perceived as part of the palliative care continuum’,^54^ and legalisation was accompanied by better financial support for palliative care.^55^ There is similar, albeit less conclusive, evidence of the same phenomenon in Oregon;^56^ and in Quebec, legislation expressly states that a right to palliative care is included in a person's right to end‐of‐life choices.^57^

The second worry is that assisted dying laws will undermine trust between patients and healthcare professionals. In reports to the U.K. House of Lords, representatives of the Royal College of General Practitionerse and the British Medical Association said that ‘several of [our] colleagues feel that there would be a significant erosion of trust in the doctor/patient relationship’, and ‘legalising assisted suicide would affect some patients' ability to trust doctors and to trust medical advice’.^58^

Again, evidence from jurisdictions with assisted dying laws should reassure us about these worries. The Netherlands (with the most permissive assisted dying laws) is characterized by the highest levels of trust for doctors, and by the best communication between doctors and their patients concerning end‐of‐life decisions.^59^ In Oregon, legalization has helped doctors to discuss all of their patients' concerns and requests, including the desire to die, thereby reducing the danger that patients feel abandoned and distressed at a time of great vulnerability.^60^ Polling suggests that few patients would come to distrust their doctors if assisted dying were legalized.^61^

So, there is no evidence that assisted dying laws have bad effects on other aspects of healthcare for people with disabilities. In fact, there is evidence that legalization goes hand in hand with increased support for palliative care, and with increased levels of trust between patients and healthcare professionals. Those effects are highly context‐dependent: legalizing assisted dying alone will not deal with wider problems of funding for disability support or palliative care. Proponents of assisted dying often also advocate better care funding for the very same reasons as they advocate legal change.^62^ But the evidence suggests that, at the very least, assisted dying laws do not undermine those important provisions.

## 6 CONCLUSION

Some people reject assisted dying laws on the grounds that people with disabilities are univocal in their fear or opposition to such laws. This article has shown that this is a mistake. The data show that there is no consensus amongst disabled rights organizations on the question of assisted dying, and that people with disabilities defend the same diversity of opinions as their fellow citizens. This is as one would expect with a complex ethical matter.

What we should do instead is show respect for disabled voices by engaging with the reasons they articulate for and against assisted dying. The conclusion of this article is that the reasons offered do not undermine the case for assisted dying. In fact, they do the opposite. If we are interested in minimizing harm, then the status quo causes much suffering that assisted dying laws might prevent. If we are concerned with respect for people with disabilities, then the case is strong for expressing it through facilitating autonomy in this key domain. If we are worried about the consequences for the wider healthcare system, we should be reassured by evidence that support for assisted dying can go along with greater trust and greater support for palliative care.

This is not to dismiss the concerns explored in this article. They pick out dangers that a properly conceived assisted dying law must seek to avert through safeguards that maximize protection while also respecting individual autonomy. Designing safeguards by drawing on disabled perspectives can build confidence (amongst people with disabilities and also amongst the wider public) that assisted dying laws take the lived experience of vulnerability into proper account.

This article concludes with two final remarks on what it means for that process of designing safeguards to be successful. The first is that we should apply here the same standard as we apply to other medical domains with similarly high stakes: a rational evaluation of the risks of change compared with the ongoing harms of the status quo. As Riddle puts it, although there is risk, ‘the potential for harm reduction is greater’, and:

the experiences relayed by people with disabilities and the words of caution expressed are valuable in assessing the system to reduce or eliminate the possibilities of harm, but not to eliminate or prevent the system itself.^63^

This means that it is a mistake to think that proponents of assisted dying must demonstrate that legalization carries no risks of abuse whatsoever. That would be inconsistent with what we do in all other risky areas of public policy, and would ignore the central point that the status quo is intolerable because it *necessitates* ongoing harms not lesser than the *possible* abuses of assisted dying laws.

The second remark is to do with context. People with disabilities continue to face social stigma, inequalities in access to public life, and a lack of adequate support for basic social, economic and civic participation. Those problems need urgent attention, but it would be a mistake to conclude that we should oppose legalizing assisted dying until they are fixed.^64^ That would be to ignore the ongoing harms of the status quo. Also, assisted dying laws, perhaps precisely by drawing attention to that wider context, can prompt improvements to other aspects of end‐of‐life care, as we saw in Section 4 above. There is no tension between assisted dying and a well‐supported regime of palliative care for those patients who do not seek to end their lives.

So, opposing assisted dying on the grounds that we must first eliminate disability‐based injustice is a mistake. The point generalizes. All arguments considered in this article share an air of paradox. We have seen appeals to the views of people with disabilities that mistake what those views actually are, and exhortations of respect that undermine the very things (well‐being, autonomy, and consent) that genuine respect puts at the heart of end‐of‐life policy. Whatever we think of other reasons someone might have for opposing assisted dying laws—there are religious, moral and political arguments we have not touched on here—it is clear that considerations of disability offer no support for that position.

## CONFLICT OF INTEREST

The author declares no conflict of interest.

## Footnotes

1

L. P. Francis , & A. Silvers (2014). Disability and assisted death. In J. D Arras, E. Fenton & R. Kukla (Eds.), *The Routledge Companion to Bioethics* (pp. 486–499). Routledge.

2
For example, Wallace, C. (2017, Sep 27). Euthanasia a choice for people with disability? It's a threat to our lives. *The Guardian*. Retrieved from: https://www.theguardian.com/commentisfree/2017/sep/27/euthanasia-a-choice-for-people-with-disability-its-a-threat-to-our-lives [Accessed Feb 15, 2022]. Hale, J. (2018, Jun 1). We're told we are a burden. No wonder disabled people fear assisted suicide. *The Guardian*. Retrieved from: https://www.theguardian.com/commentisfree/2018/jun/01/disabled-people-assisted-dying-safeguards-pressure [Accessed Feb 15, 2022]; Fraser, M. (2021, Jul 27). Assisted suicide is at odds with Western Christian tradition of human rights. The Scotsman. Retrieved from: https://www.scotsman.com/news/opinion/columnists/assisted-suicide-is-at-odds-with-western-christian-tradition-of-human-rights-murdo-fraser-msp-3324924 [Accessed Feb 15, 2022].

3
Reported in Shakespeare, T. (2016). Just what is the disability perspective on disability? *Hastings Center Report, 46*, 31–32.10.1002/hast.58727150416

4

G. Box , & K. Chambaere . (2021). Views of disability rights organisations on assisted dying legislation in England, Wales and Scotland: An analysis of position statements. Journal of Medical Ethics, 47, e64. 10.1136/medethics-2020-107021 33402428

5
Disability Rights UK. (2015). *Our position on the proposed Assisted Dying Bill*. Retrieved from: https://www.disabilityrightsuk.org/news/2015/september/our-position-proposed-assisted-dying-bill [Accessed Jul 30, 2021].

6
Shakespeare, op. cit. note 3, p. 31.

7
Ibid. For similar studies from the United States, see Hwang, K. (2005). Attitudes of persons with physical disabilities toward physician‐assisted death: An exploratory assessment of the vulnerability argument. *Journal of Disability Policy Studies 16*, 16–21; Tucker, K. L. (2017). Building bridges between the Civil Rights Movements of people with disabilities and those with terminal illness. *University of Pittsburgh Law Review, 78*, 329–350. The same picture emerges in a recent Canadian poll; see Ipsos. (2021). *Support for medically‐assisted dying in Canada*. Retrieved from: https://d3n8a8pro7vhmx.cloudfront.net/dwdcanada/pages/4709/attachments/original/1614267558/DWD_Canada_MAID_Feb_2021.pdf?1614267558 [Accessed Feb 15, 2022].

8
Fadem, P., Minkler, M., Perry, M., Blum, K., Moore Jr, L. F., Rogers, J., & Williams, L. (2003). Attitudes of people with disabilities towards physician‐assisted suicide legislation: Broadening the dialogue. *Journal of Health Politics, Policy and Law, 28*, 977–1001; Drum, C. E., White, G., Taitano, G., & Horner‐Johnson, W. (2010). The Oregon Death with Dignity Act: Results of a literature review and naturalistic inquiry, *Disability and Health Journal*, 3, 3–15.10.1215/03616878-28-6-97714756497

9
For example, Wallace, op. cit. note 2; Hale op. cit. note 2.

10
For example, Fraser, op. cit. note 2.

11
Box and Chambaere, op. cit. note 4, tab. 3.

12

House of Lords Select Committee on the Assisted Dying for the Terminally Ill Bill . (2005). Assisted Dying for the Terminally Ill Bill [HL] *Report* (I, p. 20). The Stationary Office.

13
For example, Doerflinger, R. (1989). Assisted suicide: Pro‐choice or anti‐life? *Hastings Center Report 19*, 16–19; Pellegrino, E. (1992). Doctors must not kill. *Journal of Clinical Ethics, 3*, 95–102; Bickenbach, J. (1998). Disability and life‐ending decisions. In M. P. Battin, R. Rhodes & A. Silvers (Eds.), *Physician assisted suicide: Expanding the debate* (pp. 123–132). Routledge; Keown, J. (2002). *Euthanasia, ethics and public policy: An argument against legalisation*. Cambridge University Press; a number of submissions to House of Lords Select Committee on the Assisted Dying for the Terminally Ill Bill, op. cit. note 12; Gill, C. J. (2010). No, we don't think our doctors are out to get us: Responding to the straw man distortions of disability rights arguments against assisted suicide. *Disability and Health Journal, 3*, 31–38; National Council on Disability. (2019). *The Danger of Assisted Suicide Laws: Part of the Bioethics and Disability Series*. National Council on Disability; Fraser, op cit. note 2.

14

D. Scoccia (2020). The disability case against assisted dying. In A. Cureton & D. Wasserman (Eds.), *The Oxford Handbook of Philosophy and Disability* (pp. 277–295, 286). Oxford University Press.

15
Bickenbach, op. cit. note 13, p. 127.

16

J. A. Rietjens , R. Deschepper , R. Pasman , & L. Deliens . (2012). Medical end‐of‐life decisions: Does its use differ in vulnerable patient groups? A systematic review and meta‐analysis. Social Science and Medicine, 74, 1282–1287.2240164410.1016/j.socscimed.2011.12.046

17
Ibid: 1286.

18

N. Steck , M. Egger , M. Maessen , T. Reisch , & M. Zwahlen (2013). Euthanasia and assisted suicide in selected European countries and US states: Systematic literature review. Medical Care, 51, 938–944.2392940210.1097/MLR.0b013e3182a0f427

19

E. J. Emanuel , B. D. Onwuteaka‐Philipsen , J. W. Urwin , & J. Cohen (2016). Attitudes and practices of euthanasia and physician‐assisted suicide in the United States, Canada, and Europe. Journal of the American Medical Association, 316, 79–90.2738034510.1001/jama.2016.8499

20
Ibid: 79.

21
Ibid: 87.

22

M. P. Battin , A. van der Heide , L. Ganzini , G. van der Wal , & B. D. Onwuteaka‐Philipsen (2007). Legal physician‐assisted dying in Oregon and the Netherlands: Evidence concerning the impact on patients in “vulnerable” groups. Journal of Medical Ethics, 33, 591–597, 591.1790605810.1136/jme.2007.022335PMC2652799

23
Ibid: 594.

24
Ibid: 597.

25

I. G. Finlay , & R. George (2011). Legal physician assisted suicide in Oregon and the Netherlands: Evidence concerning the impact on patients in vulnerable groups—another perspective on Oregon's data. Journal of Medical Ethics, 37, 171–174; Canetto, S., & McIntosh, J. L. (2021). A comparison of physician‐assisted/Death‐With‐Dignity‐Act death and suicide patterns in older adult women and men. *American Journal of Geriatric Psychiatry, 30*(2), 211–220. 10.1016/j.jagp.2021.06.003.21071568

26

S. Dierickx , L. Deliens , J. Cohen , & K. Chambaere (2015). Comparison of the expression and granting of requests for euthanasia in Belgium in 2007 v 2013. JAMA Internal Medicine, 175, 1703–1706.2625911310.1001/jamainternmed.2015.3982

27
Contrary to what some commentators have said, for example, Baroness Finlay in Davis, J., & Finlay, I. (2015). Would judicial consent for assisted dying protect vulnerable people? *BMJ 351*, h4437.10.1136/bmj.h443726294757

28
Dierickx et al., op. cit. note 26, pp. 1704–1705.

29
Ibid. Citing Cohen, J., Van Landeghem, P., Carpentier, N., & Deliens, N. (2013). Different trends in euthanasia acceptance across Europe: A study of 13 western and 10 central and eastern European countries, 1981–2008. *European Journal of Public Health, 23*, 378–380.10.1093/eurpub/cks18623275485

30

F. Norwood , G. Kimsma , & M. P. Battin (2009). Vulnerability and the ‘slippery slope’ at the end‐of‐life: A qualitative study of euthanasia, general practice and home death in the Netherlands. Family Practice, 26, 472–480.1982857310.1093/fampra/cmp065

31

M. C. Snijdewind , D. L. Willems , L. Deliens , B. D. Onwuteaka‐Philipsen , & K. Chambaere (2015). A study of the first year of the end‐of‐life clinic for physician‐assisted dying in the Netherlands. JAMA Internal Medicine, 175, 1633–1640.2625853410.1001/jamainternmed.2015.3978

32

K. Hedberg , & C. New (2017). Oregon's Death With Dignity Act: 20 years of experience to inform the debate. Annals of Internal Medicine, 167, 579–583.2897523210.7326/M17-2300

33
van Wesemael, Y., Cohen, J., Bilsen, J., Smets, T., Onwuteaka‐Philipsen, B., & Deliens, L. (2011). Process and outcomes of euthanasia requests under the Belgian Act on Euthanasia: A nationwide survey. *Journal of Pain and Symptom Management*, 721–733, 731. The study corroborates what was found in Smets, T., Bilsen, J., Cohen, J., Rurup, M. L., & Deliens, L. (2010). Legal euthanasia in Belgium: Characteristics of all reported euthanasia cases. *Medical Care, 48*, 187–192.10.1016/j.jpainsymman.2011.02.01121570807

34

N. Steck , C. Junker , M. Maessen , T. Reisch , M. Zwahlen , M. Egger , for the Swiss National Cohort. (2014). Suicide assisted by right‐to‐die associations: A population based cohort study. International Journal of Epidemiology, 43, 614–622.2455025010.1093/ije/dyu010

35
Downar, J., Fowler, R. A., Halko, R., Huyer, L. D., Hill, A. D., & Gibson, J. L. (2020). Early experience with medical assistance in dying in Ontario, Canada: A cohort study. *Canadian Medical Association Journal, 192*, E173–E181; Redelmeier, D., Ng, K., Thiruchelvam, D., & Shafir, E (2021). Association of socioeconomic status with medical assistance in dying: A case‐control analysis. *BMJ Open, 11*, e043547.10.1503/cmaj.200016PMC704382232051130

36
For example, Krahn, G. L. (2010). Reflections on the debate on disability and aid in dying. *Disability and Health Journal, 3*, 51–55; Campbell, J. (2019). Disabled people like me fear legal assisted suicide: It suggests that some lives are less worth living. *BMJ Opinion*. Retrieved from: https://blogs.bmj.com/bmj/2019/02/06/disabled-people-like-me-fear-legal-assisted-suicide-it-suggests-that-some-lives-areless-worth-living [Accessed Jul 30, 2021.]; Scoccia, op. cit. note 14; Fraser, op. cit. note 2.10.1016/j.dhjo.2009.10.00221122768

37
Bickenbach, op. cit. note 13, p. 127. See also Fadem et al., op. cit. note 8, pp. 993–994; Drum et al., op. cit. note 8, pp. 9–10; Hendry, M., Pasterfield, D., Lewis, R., Carter, B., Hodgson, D., & Wilkinson, C. (2012). Why do we want the right to die? A systematic review of the international literature on the views of patients, carers and the public on assisted dying. *Palliative Medicine, 27*, 13–26.10.1177/026921631246362323128904

38
For example, Brock, D. (1992). Voluntary assisted euthanasia. *Hastings Center Report, 22* 10–22; Dworkin, R. (1993). *Life's Dominion*. HarperCollins; Beauchamp, T. (2006). The right to die as the triumph of autonomy. *Journal of Medical Philosophy, 31*, 643–654; Riddle, C. A. (2017). Assisted dying & disability. *Bioethics*, 31, 484–489; Sumner, L. W. (2017). Death, disability, and self‐determination. In C. A. Riddle (Ed.), *From Disability Theory to Practice: Essays in Honor of Jerome E. Bickenbach* (pp. 101–120). Lexington Books/Rowman & Littlefield; Colburn, B. (2020). Autonomy, voluntariness, and assisted dying. *Journal of Medical Ethics, 46*, 316–319.

39

B. Colburn (2010). *Autonomy and Liberalism* (pp. 77–82). Routledge.

40

Quoted in Delamothe, T . (2009). Assisted dying: What's disability got to do with it? BMJ, 339, b3446.1971012710.1136/bmj.b3446

41
Riddle, op. cit. note 38, p. 487.

42
Ibid: 488.

43
Fadem et al., op. cit. note 8, pp. 987–988.

44
Drum et al., op. cit. note 8, pp. 10–11.

45

L. J. Nelson (2003). Respect for the developmentally disabled and forgoing life‐sustaining treatment. Mental Retardation and Developmental Disabilities Research Reviews, 9, 3–9.1258713110.1002/mrdd.10055

46
Silvers, A. (1998). Protecting the innocent from physician‐assisted suicide. *Western Journal of Medicine, 166*(6), 407–409; Battin et al., op. cit. note 22, pp. 133–148.

47
Sumner, op cit. note 38, p. 19.

48
Bickenbach, op. cit. note 13, p. 128, and see also Scoccia, op. cit. note 14.

49

I. Tuffrey‐Wijne , L. Curfs , I. Finlay , & S. Hollins (2018). Euthanasia and assisted suicide for people with an intellectual disability and/or autism spectrum disorder: An examination of nine relevant euthanasia cases in the Netherlands (2012–2016). BMC Medical Ethics, 19(1), 17.2950651210.1186/s12910-018-0257-6PMC5838868

50

See, for example, Bekkema, N., de Veer, A. J. E., Hertogh, C. M. P. M., & Francke, A. L. (2013). Respecting autonomy in the end‐of‐life care of people with intellectual disabilities: A qualitative multiple‐case study. Journal of Intellectual Disability Research, 58, 368–380.2351001110.1111/jir.12023

51

See, for example, Colburn (2010), op. cit. note 39; Quong, J. (2010). Liberalism Without Perfection. Oxford University Press.

52

J. Teno , & J. Lynn (1991). Voluntary active euthanasia: The individual case and public policy. *Journal of American Geriatric Society, 39*, 827–830; Miller, R. (1992). Hospice care as an alternative to euthanasia.*Law, Medicine & Health Care, 20*, 127–132.10.1111/j.1532-5415.1991.tb02707.x2071815

53
Health and Sport Committee of the Scottish Parliament. (2015). *Stage 1 Report on Assisted Suicide (Scotland) Bill*. Scottish Parliament Corporate Body, p. 11; see Ibid: 9–10 for similar points.

54
Dierickx et al., op. cit. note 26; Berghe, P. V., Mullie, A., Desmet, M., & Huysmans, G. (2013). Assisted dying – The current situation in Flanders: Euthanasia embedded in palliative care. *European Journal of Palliative Care, 20*, 266–272.

55
Smets et al., op. cit. note 33.

56

L. Ganzini , H. D. Nelson , T. A. Schmidt , D. F. Kraemer , M. A. Delorit , & M. A. Lee (2000). Physicians' experiences with the Oregon Death with Dignity Act. New England Journal of Medicine, 342, 557–563.1068491510.1056/NEJM200002243420806

57
Statute of Quebec. (2021). *Act Respecting End‐of‐Life Care*. Quebec Official Publisher. Retrieved from: https://www.legisquebec.gouv.qc.ca/en/pdf/cs/S-32.0001.pdf [Accessed Feb 15, 2022].

58
House of Lords Select Committee on the Assisted Dying for the Terminally Ill Bill, op cit. note 12, pp. 40, 42, and 79. See also Silvers, A. (1997). Protecting the innocents: People with disabilities and physician‐assisted dying. *Western Journal of Medicine, 166*, 407–409; Snyder, L., Leffler, C., & American College of Physicians. (2005). Ethics manual. (7), *Annals of Internal Medicine, 142*, 560–582; Health and Sport Committee of the Scottish Parliament, op. cit. note 53, p. 35.

59
A. van der Heide, L Deliens, K Faisst, T Nilstun, M Norup, E. Paci, G van der Wal, & P.J. van der Maas. (2003). End‐of life decision‐making in six European countries: descriptive study. *The Lancet*, *362*, 345–350.10.1016/S0140-6736(03)14019-612907005

60
Dobscha, S. K., Heintz, R. T., Press, N., & Ganzini, L. (2004). Oregon physicians' responses to requests for assisted suicide: A qualitative study. *Journal of Palliative Medicine, 7*, 451–461; Ganzini, L., Nelson, H. D., Lee, M. A., Kraemer, D. F., Schmidt, T. A., & Delorit, M. A. (2001). Oregon physicians' attitudes about and experiences with end‐of‐life care since passage of the Oregon Death with Dignity Act. *Journal of the American Medical Association, 285*, 2363–2369.

61
For example, Graber, M. A., Levy, B. I., Weir, R. F., & Oppliger, R. A. (1996). Patients' views about physician participation in assisted suicide and euthanasia. *Journal of General Internal Medicine, 11, 71–76*. See also testimony from Tom Shakespeare and Raymond Tallis quoted in House of Lords Select Committee on the Assisted Dying for the Terminally Ill Bill, op. cit. note 12, p. 51 and passim.10.1007/BF025995818833013

62
For example, Colburn (2020), op. cit. note 38.

63
Riddle, op. cit. note 38, p. 489.

64

For example, Stainton, T . (2019). Disability, vulnerability and assisted death: Commentary on Tuffrey‐Wijne, Curfs, Finlay and Hollins. BMC Medical Ethics, 20, 89.3177572210.1186/s12910-019-0426-2PMC6882029

